# Determinants of health-related quality of life across the spectrum of connective tissue diseases using latent profile analysis: results from the LEAP cohort

**DOI:** 10.1093/rheumatology/keac680

**Published:** 2022-12-19

**Authors:** Sarah Dyball, John A Reynolds, Ariane L Herrick, Sahena Haque, Hector Chinoy, Ellen Bruce, Sophia Naz, Ben Parker, Ian N Bruce

**Affiliations:** Centre for Epidemiology Versus Arthritis, Division of Musculoskeletal and Dermatological Sciences, Faculty of Biology, Medicine and Health, University of Manchester, Manchester Academic Health Science Centre, Manchester, UK; Kellgren Centre for Rheumatology, Manchester University Hospitals NHS Foundation Trust, Manchester, UK; Rheumatology Research Group, Institute of Inflammation and Ageing, College of Medical and Dental Sciences, University of Birmingham, Birmingham, UK; Rheumatology Department, Sandwell and West Birmingham NHS Trust, Birmingham, UK; National Institute for Health Research Manchester Biomedical Research Centre, Manchester University NHS Foundation Trust, University of Manchester, Manchester, UK; Department of Rheumatology, Salford Care Organisation, Northern Care Alliance NHS Foundation Trust, Manchester Academic Health Science Centre, Salford, UK; Department of Rheumatology, Manchester University Foundation Trust, Wythenshawe Hospital, Manchester, UK; Centre for Epidemiology Versus Arthritis, Division of Musculoskeletal and Dermatological Sciences, Faculty of Biology, Medicine and Health, University of Manchester, Manchester Academic Health Science Centre, Manchester, UK; National Institute for Health Research Manchester Biomedical Research Centre, Manchester University NHS Foundation Trust, University of Manchester, Manchester, UK; Department of Rheumatology, Salford Care Organisation, Northern Care Alliance NHS Foundation Trust, Manchester Academic Health Science Centre, Salford, UK; Kellgren Centre for Rheumatology, Manchester University Hospitals NHS Foundation Trust, Manchester, UK; Department of Rheumatology, Pennine Acute Hospitals NHS Trust, Manchester, UK; Kellgren Centre for Rheumatology, Manchester University Hospitals NHS Foundation Trust, Manchester, UK; National Institute for Health Research Manchester Biomedical Research Centre, Manchester University NHS Foundation Trust, University of Manchester, Manchester, UK; Centre for Epidemiology Versus Arthritis, Division of Musculoskeletal and Dermatological Sciences, Faculty of Biology, Medicine and Health, University of Manchester, Manchester Academic Health Science Centre, Manchester, UK; Kellgren Centre for Rheumatology, Manchester University Hospitals NHS Foundation Trust, Manchester, UK; National Institute for Health Research Manchester Biomedical Research Centre, Manchester University NHS Foundation Trust, University of Manchester, Manchester, UK

**Keywords:** QoL, SLE, UCTD, PROMS, SF-36

## Abstract

**Objectives:**

Poor health-related quality of life (HRQoL) is well recognized in patients with CTD. We hypothesized that subgroups of patients across the spectrum of CTD experience different HRQoL patterns and aimed to determine patient-level characteristics associated with these different subgroups.

**Methods:**

Using the eight continuous domains of the Medical Outcomes Study 36-item Short Form (SF-36) questionnaire we performed data-driven clustering to derive latent profiles (LPs) of patients with distinct HRQoL patterns. Multivariable ordinal logistic regression was used to determine patient-level characteristics associated with each HRQoL subgroup identified.

**Results:**

A total of 309 CTD patients completed the SF-36 questionnaire. The most impaired SF-36 domains in each disease group were vitality, general health and bodily pain. The physical component of the SF-36 was consistently more impaired compared with the mental component, with similar scores across disease groups. Three LPs were identified with poor [*n* = 89 (29%)], average [*n* = 190 (61.4%)] and excellent [*n* = 30 (9.7%)] HRQoL. LPs were not associated with diagnostic grouping or autoantibody profiles. Black background [odds ratio (OR) 0.22 (95% CI 0.08, 0.63)], Indo-Asian background [OR 0.39 (95% CI 0.19, 0.78)], concomitant fibromyalgia [OR 0.40 (95% CI 0.20, 0.78)], sicca symptoms [OR 0.56 (95% CI 0.32, 0.98)] and multimorbidity [Charlson Comorbidity Index; OR 0.81 (95% CI 0.67, 0.97)] were associated with the ‘poor’ HRQoL LP.

**Conclusion:**

Distinct HRQoL subgroups exist that are not primarily driven by a specific diagnosis or autoantibody profiles. We identified a number of key demographic and clinical factors associated with poor HRQoL. These factors need to be addressed across the whole CTD spectrum as part of a holistic management approach aimed at improving overall patient outcomes.

Rheumatology key messages

- CTD patients can be clustered into distinct HRQoL subgroups that are not driven by diagnosis.
- Ethnicity, smoking, multimorbidity and sicca syndrome are key clinical predictors of poor HRQoL.
- These factors should be addressed across the whole CTD spectrum as part of holistic management.

## Introduction

Health-related quality of life (HRQoL) is a multidimensional concept representing an individual’s perception of health, encompassing spiritual, functional, physical, emotional and social well-being. Reduced HRQoL in CTDs, also known as systemic autoimmune rheumatic diseases, is likely multifactorial and may relate to organ damage and physical disability, medication adverse events, mental health and pain, as well as impaired work-related productivity and social functioning. Major goals of managing patients with a rheumatic disease are to minimize functional loss and damage, maintain independence and preserve HRQoL [1, 2].

Poor HRQoL is recognized in patients with established CTDs, however, it is unclear how it affects patients with UCTD, which has traditionally been associated with a ‘mild’ or more benign profile [3, 4]. The Medical Outcomes Study 36-item Short Form (SF-36) questionnaire is the most commonly used, comprehensive and generic HRQoL measure and has been validated in rheumatic diseases including SLE, SSc, idiopathic inflammatory myopathies (IIMs) and primary SS (pSS) [5–9].

Latent profile analysis (LPA) is an exploratory, data-driven, statistical method for grouping individuals based on shared features, but it is a novel approach in research of HRQoL in rheumatic diseases. Current research into HRQoL among patients with CTDs has predominately looked at factors associated with HRQoL in a single disease or performed a comparison of several rheumatic diseases; however, there have been no studies investigating whether across CTDs there are subgroups of patients who have shared HRQoL profiles. LPA can identify subpopulations within a group of individuals based on a number of variables [10]. This method lends itself to the multidimensional nature of HRQoL by allowing us to identify whether there are distinct groups of people with similar patterns of QoL responses in terms of the domains affected. This can identify those individuals who are most in need of intervention or areas to target to improve HRQoL.

We hypothesized that subgroups of patients across the spectrum of CTDs experience different HRQoL patterns, irrespective of their clinical diagnosis. Our objectives were to investigate HRQoL in patients affected with a variety of CTDs (including UCTD) using the SF-36, to identify subgroups of patients who experience shared HRQoL patterns across a CTD diagnosis and to determine patient-level characteristics associated with different subgroups of HRQoL identified.

## Patients and methods

### Study population

Between May 2014 and June 2019, adult patients were recruited into the Lupus Extended Autoimmune Phenotype (LEAP) cohort from Manchester University NHS Foundation Trust (three sites) and the Northern Care Alliance NHS Foundation Trust (two sites). Patients with an established CTD diagnosis and clinically stable disease were eligible for inclusion if they had one or more clinical feature of a CTD and one or more antibody within the ANA spectrum. Autoantibodies were measured historically, according to physician discretion, but all included the BioPlex 2200 ANA Screen [11]. Rheumatologist diagnosis was used to classify patients into four groups: SLE, pSS, UCTD and those with IIM, SSc or an overlap syndrome including MCTD (combined due to low numbers in these CTD subtypes).

Ethical approval was obtained from the Greater Manchester East Research Ethics Committee (13/NW/0564), all patients signed written informed consent and the study was conducted in accordance with the Declaration of Helsinki.

### Measurement of HRQoL

The SF-36 version 2 QoL questionnaire was completed at enrolment and includes eight domains: physical function (PF), role physical (RP), bodily pain (BP), general health (GH), vitality (VT), social functioning (SF), role emotional (RE) and mental health (MH), with scores ranging from 0 to 100, with higher scores reflecting better HRQoL. The physical component summary (PCS) and mental component summary (MCS) scores were calculated for each group, with a score <50 representing a worse HRQoL compared with the general UK population [12]. All patients completed the SF-36 questionnaire and missing values were imputed using methodology suggested by the SF-36 manual [13].

The European Quality of Life 5-dimension 3-level (EQ-5D-3L) questionnaires were completed in a subgroup of the cohort (those recruited after 20 June 2016) concurrently with the SF-36 questionnaire. The EQ-5D-3L is a generic HRQoL measure that can be used for cost-effectiveness studies across diseases [14, 15]. The EQ-5D-3L index score ranges from 1 (full health) to <0 (worse than being dead) and is calculated from five descriptive questions that encompass five domains of HRQoL: mobility, self-care, usual activities, pain/discomfort and anxiety/depression. The EuroQol visual analogue scale (EQ-VAS) reports self-rated health (score range 0–100) from ‘worst imaginable health state’ to ‘best imaginable health state’.

### Covariates

Demographic factors, smoking status, medical records-confirmed comorbid conditions and previous rheumatic therapies were recorded. Comorbidities were categorized using the Charlson Comorbidity Index (CCI) [16]. Missing data were collected where possible from patient records.

### IFN-stimulated gene (ISG) scores

ISG scores were calculated as previously described using a six-gene reverse transcription quantitative polymerase chain reaction (RT-qPCR) analysis [17]. The mean ISG was calculated using data from healthy controls, with scores >2 s.d. above the mean calculated and scores above this value (>2.466) designated as positive.

### Statistical analysis

Descriptive statistics were calculated using Stata version 14 (StataCorp, College Station, TX, USA) and R version 4.0.4 (R Foundation for Statistical Computing, Vienna, Austria) using Kruskal–Wallis or chi-squared testing to compare across the four disease groups, unless specified otherwise. Spearman’s *r* was used to assess correlation. Univariate linear and logistic regression models were used with SLE as the referent group. Multivariable models included all covariates with a *P*-value <0.1 unless otherwise specified. A *P*-value <0.05 was considered statistically significant.

### LPA models

LPA is a statistical method for identifying homogeneous subgroups of individuals based on a set of continuous measured variables. Classification of individuals into latent classes is probabilistic and LPA allows for selection of the optimum numbers of classes, through comparison of model fit indices. Using the eight continuous domains of the SF-36 questionnaire, we used the *mclust* model-based clustering package in R version 4.0.4 to implement a more generalized version of LPA to identify clusters of patients who experienced distinct HRQoL patterns [18]. In all models, variances were equated and covariances fixed to zero. We used the Bayesian Information Criteria (BIC), Integrated Completed Likelihood (ICL) criterion and bootstrap sequential likelihood ratio test (LRT) to compare fits of models with different covariance structures and numbers of LPs. Demographic, clinical and serological variables were compared between clusters. Further details of LPA methods are given in Supplementary Data S1, available at *Rheumatology* online.

### Predicting LP membership

Multivariable ordered logistic regression models were used to determine whether patient-level characteristics were significantly associated with LP membership, with the lowest HRQoL LP (LP1) as the referent group. The proportional odds assumption was tested using the Brant test and maximum likelihood ordered logit estimation [19]. Multicollinearity was tested using the variance inflation factor (VIF).

## Results

### Baseline demographics

Data were collected from 309 patients {280 (90.6%) women, median age 51 years [interquartile range (IQR) 40–59]}: 237 (76.7%) patients were Caucasian, 46 (14.9%) were of Black background and 18 (5.8%) were of Indo-Asian background. By rheumatologist diagnosis, 115 (37.2%) had SLE, 56 (18.1%) had pSS, 72 (23.3%) had UCTD and 66 (21.4%) had SSc-IIM spectrum disorder; this fourth group consisted of 25 patients with SSc, 28 with an overlap syndrome and 13 with IIM. Supplementary Table S2, available at *Rheumatology* online, reports the demographic data of patients with SSc, IIM and overlap syndromes.

Patients with an SSc-IIM spectrum disease or pSS were older than those with SLE. Disease duration differed across diagnostic groups (kwallis, *P* < 0.001), with the longest disease duration being in patients with SLE [median 11.0 years (IQR 5.4–18.0)]. There were 101 (32.7%) patients with a CCI score of zero, 150 (48.5%) patients with a score of 1–2 and 58 (18.8%) patients with a score >3. The most common chronic comorbidities included reflux disease [*n* = 48/309 (15.5%)] thyroid disease [46/309 (14.9%)] and respiratory disease [42/309 (13.6%)]. Peptic ulcer disease and respiratory disease were most common in SSc-IIM spectrum disease [14/66 (21%) and 16/66 (24%), respectively) as shown in Supplementary Table S3, available at *Rheumatology* online. There were 42 (13.6%) patients with coexisting fibromyalgia, 25 (8.1%) with hypermobility and 20 (6.5%) with anxiety or depression. The rate of fibromyalgia was highest in pSS [*n* = 11/56 (20%)] and hypermobility was greatest in UCTD [*n* = 10/72 (15%)]. Patients with SSc-IIM disease had the lowest rates of anxiety and depression [*n* = 2/66 (3%)].

Prior oral steroid and immunosuppressant use was highest in SLE (82% and 59%, respectively) and SSc-IIM spectrum disease (59% and 52%, respectively). A proportion of patients from all disease groups had been prescribed biologic therapies, but this was highest in SLE and SSc-IIM spectrum disease (12% and 6%, respectively). Antihypertensive use was similar across diseases in approximately one-third of patients [*n* = 101/309 (33%)], whereas statin use was most common in SLE [*n* = 21/103 (18%)] and least common in SSc-IIM disease [*n* = 6/66 (9%)]. Table 1 shows the main demographic, clinical and therapeutic characteristics by disease group.

**Table 1.** Baseline demographics of the four diagnostic groups

Characteristics	SLE (*n* = 115)	pSS (*n* = 56)	UCTD (*n* = 72)	SSC/IIM/overlap (*n* = 66)
Age, years, median (IQR)	47 (34–54)	55.5 (44.5–60.5)	47.5 (37–58)	57.5 (50–64)
Female, *n* (%)	106 (92.2)	53 (94.6)	61 (84.7)	60 (90.9)
Disease duration, years, median (IQR) (*N* = 298)	11.0 (5.4–18.0)	3.7 (2.5–8.0)	3.8 (1.8–7.3)	6.1 (2.9–14.0)
Ethnicity, *n* (%)				
Caucasian	88 (76.5)	47 (83.9)	48 (66.7)	54 (81.8)
Indo-Asian	7 (6.1)	5 (8.9)	4 (5.6)	2 (3.0)
Black	17 (14.8)	3 (5.4)	17 (23.6)	9 (13.6)
Other	3 (2.6)	1 (1.8)	3 (4.2)	1 (1.5)
BMI, kg/m^2^, median (IQR) (*N* = 304)	26.9 (22.6–32.0)	29 (25.4–32.0)	28.5 (23.8–32.4)	28 (23.6–32.8)
Currently smoke, *n* (%) (*n* = 306)	10 (8.7)	4 (7.1)	8 (11.1)	4 (6.1)
Fibromyalgia, *n* (%)	14 (12.2)	11 (19.6)	9 (12.5)	6 (9.1)
Anxiety, *n* (%)	6 (5.2)	2 (3.6)	3 (4.2)	1 (1.5)
Depression, *n* (%)	8 (7.0)	4 (7.1)	3 (4.2)	1 (1.5)
CCI, mean (s.d.)	1.2 (1.3)	1.4 (1.1)	1.2 (1.4)	1.8 (1.2)
Medications (ever), *n* (%)				
Oral steroids (*N* = 306)	94 (81.7)	19 (33.9)	25 (34.7)	34 (51.5)
Immunosuppressants (*N* = 300)	68 (59.1)	15 (26.8)	22 (30.6)	34 (51.5)
Biologic therapy (*N* = 302)	14 (12.2)	1 (1.8)	3 (4.2)	4 (6.1)

*N* represents the total number of patients with data for this variable if complete data not available.

### HRQoL scores by diagnosis

**Figure 1.**
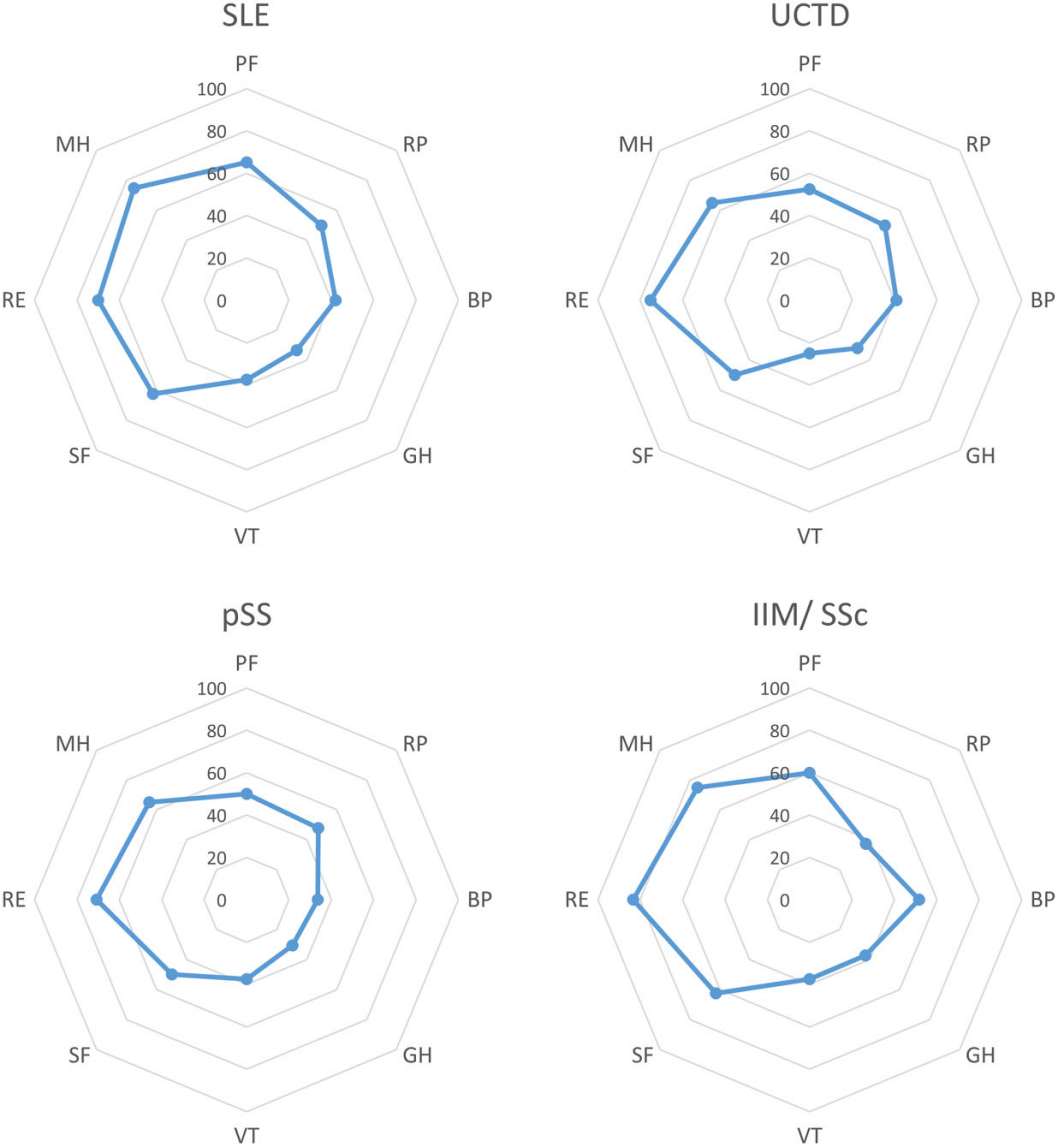
Radar diagrams of the SF-36 score in each CTD diagnostic group ranging from 0 (worst) to 100 (best) in each of the eight HRQoL domains

The eight domains of the SF-36 questionnaire are shown by diagnostic group in Fig. 1 and the most impaired domains in each disease group were VT, GH and BP. When comparing medians across groups (Supplementary Table S4, available at *Rheumatology* online), VT was significantly lower in patients with UCTD and pSS compared with SLE and IIM/SSc (kwallis, *P* = 0.005) and BP, which was higher in SLE, UCTD and pSS compared with IIM/SSc (kwallis, *P* = 0.047).

**Figure 2.**
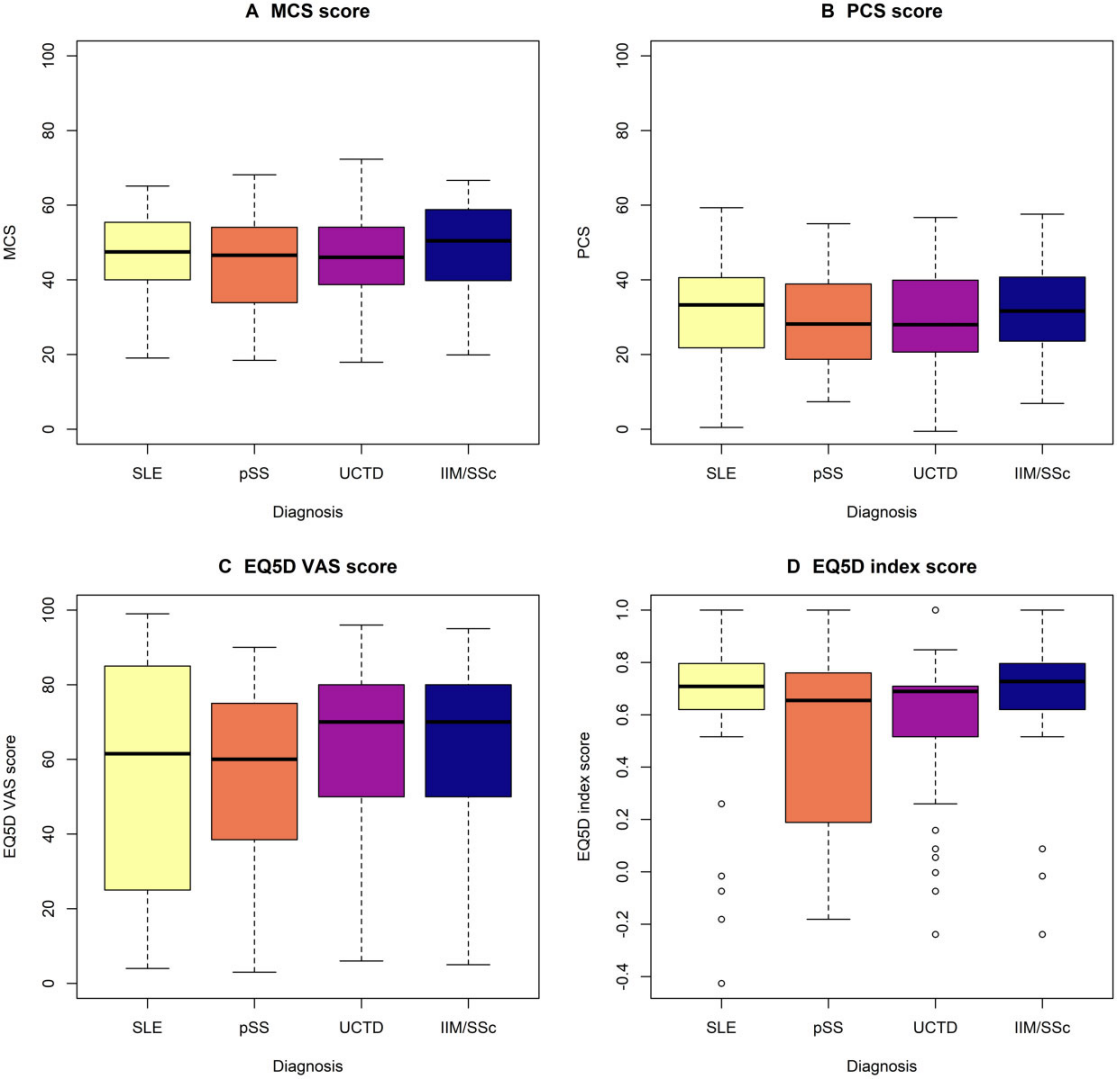
**(A)** MCS, **(B)** PCS, **(C)** EQ-VAS and **(D)** EQ-5D index score across four diagnostic groups

The PCS and MCS scores (calculated from the eight SF-36 domains) by diagnosis group are shown in Fig. 2A and B. The PCS was more impaired compared with the MCS, with similar scores across disease groups. There was wide variation in both the PCS and MCS scores across diseases. In particular, there were a significant number of patients from each disease group exhibiting MCS scores that were higher, in line or lower than the general population. The majority of patients had a PCS score lower than the average UK population.

### EQ-5D scores by diagnosis

EQ-5D scores were available in 174/309 (56.3%) patients in this cohort and are summarized in Fig. 2C and D. Pain, usual activity and mobility were the most significantly impaired domains of the EQ-5D, shown in Supplementary Fig. S7, available at *Rheumatology* online. The EQ-5D index score was lower in the pSS group compared with SLE [β = −0.14 (95% CI −0.26, −0.01), *P* = 0.038]) but was otherwise similar between groups. The EQ-VAS was not significantly different between disease groups. The SF-36 PCS correlated well with EQ-5D index score (*r* = 0.689, *P* < 0.0001) and less strongly with the EQ-VAS (*r* = 0.420, *P* < 0.0001). The MCS also correlated more strongly with the EQ-5D index score (*r* = 0.601, *P* < 0.0001) than with the EQ-VAS (*r =* 0.396, *P* < 0.0001).

### LPA

**Figure 3.**
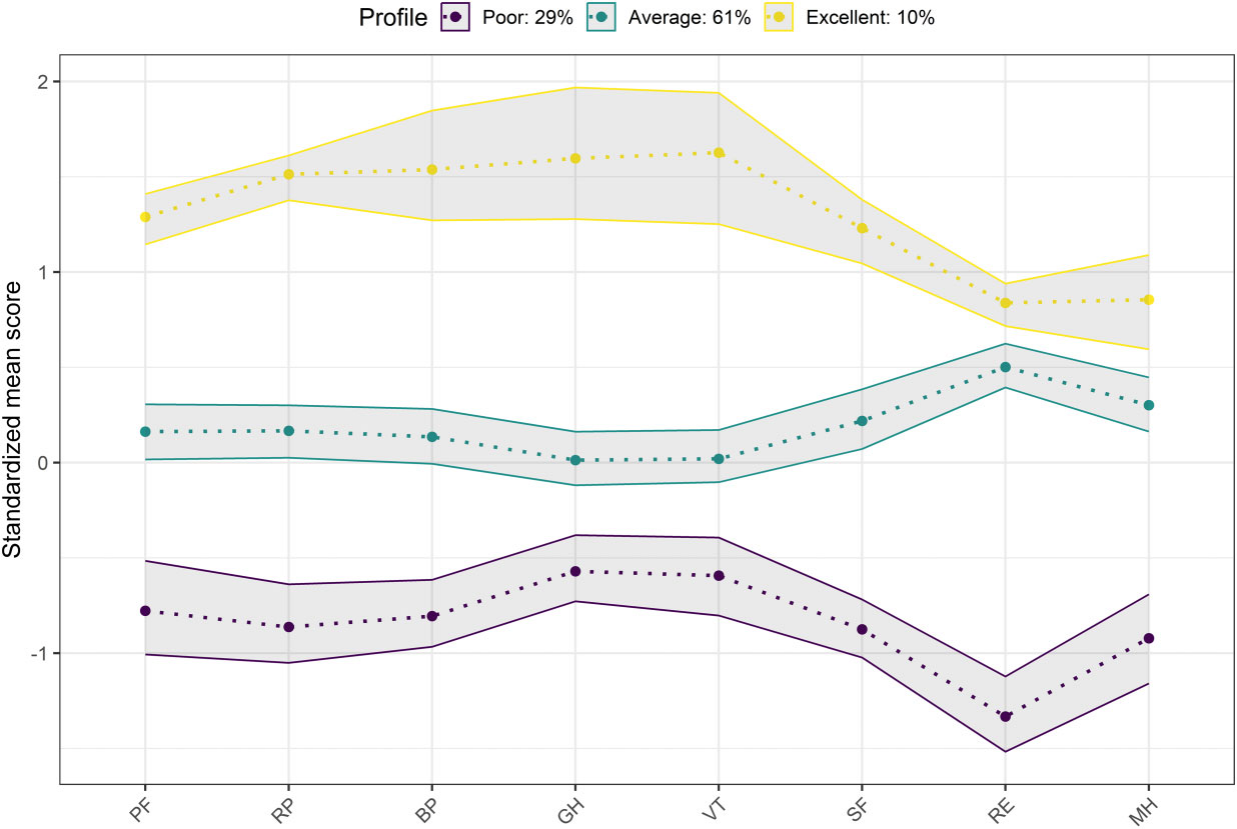
The three LPs graphically represented by mean SF-36 domain scores and 95% CI. LP1 (poor), *n* = 89 (29%); LP2 (average), *n* = 190 (61%); LP3 (excellent), *n* = 30 (10%)

HR-QoL did not differ significantly across diagnostic groups (Fig. 2), however, there was wide intragroup variation, suggesting hidden groups across diseases. An LP approach was taken that identified three HRQoL LPs (Fig. 3). The profiles had mean overall MCS scores of 34.8 (s.d. 9.0), 50.3 (s.d. 8.4) and 56.5 (s.d. 5.1), respectively, and mean PCS scores of 21.7 (s.d. 9.0), 31.6 (s.d. 11.3) and 51.5 (s.d. 4.2), respectively. LP1 [*n* = 89 (29%)] had the poorest HRQoL scores across all domains and was considered the ‘poor’ HRQoL profile. LP3 [*n* = 30 (10%)] had the highest HRQoL scores across domains and is considered the ‘excellent’ HRQoL LP. LP2 [*n* = 190 (61%)] had intermediate physical and mental well-being and was considered the ‘average’ HR-QoL profile. LPs were not associated with diagnostic groupings (Table 2).

**Table 2.** Membership of the three LPs by each clinical diagnostic group

Diagnosis	Poor (LP1) [*N* = 89 (29%)]	Average (LP2) [*N* = 190 (61%)]	Excellent (LP3) [*N* = 30 (10%)]	Overall [*N* = 309 (100%)]	*P*-value
SLE	29 (32.6)	76 (40.0)	10 (33.3)	115 (37.2)	0.440
pSS	21 (23.6)	30 (15.8)	5 (16.7)	56 (18.1)	0.281
UCTD	19 (21.3)	47 (24.7)	6 (20.0)	72 (23.3)	0.744
SSc	11 (12.4)	11 (5.8)	3 (10.0)	25 (8.1)	0.159
MCTD/overlap	5 (5.6)	18 (9.5)	5 (16.7)	28 (9.1)	0.180
IIM	4 (4.5)	8 (4.2)	1 (3.3)	13 (4.2)	0.963

Data are presented as *n* (%).

The LP matched the EQ-5D index and VAS scores. The median EQ-5D index score was 0.52 (IQR −0.02–0.65) in LP1, 0.69 (0.62–0.80) in LP2 and 1.0 (0.80–1.0) in LP3. The median VAS was 50 (IQR 35–65), 70 (46.5–80) and 89 (82–90) in each LP, respectively.

### Predicting latent profile membership

An ordinal logistic regression model was used, as the proportional odds assumption was met (Brant test *P* = 0.474, likelihood ratio test *P* = 0.539) and multicollinearity was deemed to not be an issue (VIF range 1.02–1.15).

Gender, age and disease duration were not associated with the HRQoL LP subgroup (Table 3). The poor HRQoL subgroup was associated with Black and Indo-Asian background, multimorbidity (defined using the CCI) and smoking. Sicca symptoms, but not a diagnosis of pSS or anti-Ro/La antibodies, were associated with being in the poor HRQoL LP subgroup. Steroid, immunosuppressant and biologic therapies were not associated with LP membership.

**Table 3.** Univariate and multivariable ordinal logistic regression comparing patient characteristics across LPs, with LP1 (poor HRQoL) used as the reference category

Characteristics	Univariate, OR (95% CI)	*P*-value	Multivariable, OR (95% CI)^a^	*P*-value
Demographics				
Age	0.99 (0.97, 1.00)	0.131		
Female	0.56 (0.24, 1.25)	0.157		
Disease duration	0.99 (0.95, 1.04)	0.754		
Black ethnicity	**0.28 (0.11**, **0.74)**	**0.010**	**0.12 (0.04**, **0.37)**	**<0.001**
Indo-Asian ethnicity	**0.53 (0.28**, **1.01)**	**0.056**	**0.27 (0.13**, **0.57)**	**0.001**
Obesity	**0.56 (0.35**, **0.88)**	**0.013**	0.62 (0.38, 1.03)	0.064
Cigarette smoker	**0.55 (0.34**, **0.87)**	**0.012**	**0.55 (0.32**, **0.84)**	**0.038**
Clinical diagnosis				
pSS	0.64 (0.34, 1.22)	0.176		
UCTD	0.95 (0.53, 1.71)	0.861		
SSc-IIM spectrum	0.96 (0.52, 1.78)	0.900		
Comorbidities				
Fibromyalgia	**0.40 (0.21**, **0.77)**	**0.006**	0.49 (0.23, 1.05)	0.065
Anxiety or depression	**0.38 (0.16**, **0.94)**	**0.037**	0.37 (0.10, 1.38)	0.065
CCI	**0.82 (0.69**, **0.98)**	**0.026**	**0.78 (0.63**, **0.97)**	**0.024**
Medication use (ever)				
Steroids	0.78 (0.49, 1.23)	0.280		
Immunosuppressants	1.11 (0.70, 1.76)	0.644		
Disease-related factors				
Sicca syndrome	**0.55 (0.35**, **0.88)**	**0.013**	**0.53 (0.30**, **0.94)**	**0.031**
Anti-Ro positive	0.90 (0.56, 1.44)	0.667		
Anti-dsDNA positive	**1.91 (1.11**, **3.26)**	**0.019**	1.61 (0.79, 3.27)	0.190
Low complement	1.63 (0.98, 2.70)	0.059	0.98 (0.51, 1.88)	0.955
Positive ISG score	1.03 (0.99, 1.08)	0.179		

a Multivariate analysis adjusted for variables in univariate analysis with *P* < 0.1 (ethnicity, fibromyalgia, anxiety or depression, CCI, sicca syndrome, anti-dsDNA antibodies, low complement, obesity and cigarette smoking).

Significant values in bold.

The OR is the odds of being in a higher LP (i.e. a better HRQoL) for a unit increase in each variable. For disease group, SLE was the reference; for ethnicity, white was the reference.

### ISGs

ISG analysis was performed in 159/309 (51.5%) patients; 58/159 (36.5%) patients had a positive ISG score. There was no association between the ISG score and either the MCS or PCS. There was no association between the ISG score and LP group.

## Discussion

In this mixed cohort of patients with CTD, there were no clear differences in HRQoL between different CTD diagnoses. Patients with UCTD had comparable HRQoL to patients with other CTDs. Overall, the physical components of HRQoL were consistently more impaired than the mental components. Due to the wide variation in scores across CTD diagnoses, we hypothesised that HRQoL subgroups exist independently of CTD diagnosis. Our LP analysis identified three LPs that we termed poor, average and excellent HRQoL LP; again, these LPs were not associated with diagnostic grouping. As far as we are aware, this study is the first to apply LP analysis to HRQoL outcomes in patients with a CTD.

It is clinically pertinent to rheumatologists who review the full spectrum of patients to recognize that similar factors drive HRQoL irrespective of diagnosis. Patients who were of Black or Indo-Asian background, had sicca syndrome, smoked or had multimorbidity were more likely to be in a lower HRQoL LP. Understanding which patient-level characteristics might be most associated with poor HRQoL LP membership across CTD diagnoses may help stratify patients most likely to benefit from HRQoL management strategies. Furthermore, some modifiable patient factors such as obesity and smoking status were identified as being associated with membership in a poorer HRQoL LP and could be addressed to potentially improve HRQoL.

HRQoL measurements are used in randomized controlled trials (RCTs) to determine treatment efficacy, in observational studies, as well as increasingly in clinical practice to understand the impairment in an individual patient. In landmark RCTs enrolling CTD patients, patient-reported outcome measures (PROMs), including the SF-36 and EQ-5D, are commonly used secondary outcomes [20–23], and in pSS the EULAR Sjögren’s Syndrome Patient Reported Index has been used as a primary outcome measure [24]. The US Food and Drug Administration recommends that PROMs should be used to assess efficacy in clinical trials [25], and the OMERACT group has recommended that HRQoL assessments should be considered a core measurement in RCTs [26].

Previous studies have shown that patients with a CTD have a lower HRQoL compared with the general population, as well as other chronic diseases such as diabetes and congestive heart failure [27, 28]. A major contributor to reduced HRQoL compared with the general public is the CTD diagnosis itself; in RA, worry about the consequences of illness has been shown to be a stronger correlate of physical HRQoL than pain [29]. HRQoL is complex and multifactorial. In CTDs, both physical health (e.g. disability, loss of function, pain and fatigue) and psychosocial health (e.g. fear for the future, depression and anxiety, loss of role and reduced participation in social and work-related activities) may be affected. This is the first study to show that UCTD has a PCS and MCS score in keeping with other CTDs. In this cohort, patients with UCTD were more likely to be from a non-White background or to smoke, both of which were associated with reduced HRQoL. Furthermore, we would suggest that the uncertainty around this diagnosis, and lack of understanding from support networks, may also play a role in reducing HRQoL, in line with other CTDs. There may be a disconnect in the agenda between what is important for the patient and what is important for the clinician, e.g. fatigue, pain and cognitive dysfunction are commonly reported as the most disabling symptoms for patients with a CTD, which are highly prevalent in patients with UCTD and may in part explain their poor HRQoL [30–35]. Furthermore, the invisibility of these symptoms to healthcare professionals, as well as society more widely, generates an additional challenge of feeling ‘disbelieved’ [36].

There were differences in the ethnic backgrounds of patients in each HRQoL profile but no difference in other demographic variables. The relationship between ethnicity and HRQoL is complex. Patients with Black and Indo-Asian ethnicity are more likely to have more severe disease and worse outcomes [37–39]. Furthermore, HRQoL is influenced by health inequalities, which disproportionately affect patients from ethnic minority groups, including secure employment, education level and economic status [40].

Fibromyalgia commonly manifests with non-specific pain and fatigue, and often coexists in patients with CTDs, making it difficult to disentangle from damage and CTD-related disease activity [41–43]. Furthermore, chronic pain and fatigue are often a barrier to exercise, which can improve mood and HRQoL. Further work is required to understand how early intervention with physical therapy, in conjunction with psychological and pharmacotherapies where appropriate, may improve HRQoL in these groups.

Comorbidity, in particular multimorbidity, has previously been shown to be associated with poorer HRQoL [44, 45]. As we move toward managing an ageing, multimorbid population, we should be aware of the complex health needs of this population and the link between multimorbidity and HRQoL.

All pSS patients and one-third of patients with other clinical diagnoses exhibited sicca syndrome in this cohort, which includes a proportion of patients from each diagnostic group. Sicca syndrome, but not pSS or anti-Ro antibodies, were associated with poor HRQoL. Both xerophthalmia and xerostomia may be underappreciated by clinicians, in part because they are difficult to treat, however, they can be disabling to patients and associated with negative health outcomes [30, 46]. Xerostomia is associated with a susceptibility to dental caries and periodontal disease, increases the incidence of oral candidiasis and ulceration and causes difficulties in chewing and swallowing and changes in taste perception that contribute to impaired HRQoL [47, 48]. Interestingly, although HRQoL is reduced in SS patients when compared with healthy controls, no difference was seen when compared with non-SS sicca patients [49–51], suggesting that dryness itself plays an important role in HRQoL.

Due to the ‘mixed’ nature of this cohort, no formal disease activity scores were collected. Anti-dsDNA and low complement are serological indicators of high disease activity in SLE and may be associated with a higher HRQoL LP. The ISG was measured as a molecular biomarker and potential treatment target that is prevalent across patients with CTDs, however, it did not correlate with HRQoL scores. This is in line with previous studies that showed fatigue severity does not correlate with inflammatory cytokines, including IFN [33].

CTDs frequently share common clinical manifestations, immunopathology and medications; e.g. type 1 IFN has previously been implicated across all diagnostic groups in this cohort [17]. Directly targeting molecular pathology in biomarker-positive patients could revolutionize drug development by improving response to therapy, and if regulatory approval was sought across diseases, it would benefit overlap conditions or undifferentiated diseases that would be traditionally excluded from clinical trials. A limitation of a basket trial approach includes the difficulty of measuring disease-specific outcomes in a mixed population. This analysis has demonstrated that outcomes can be measured across CTDs with distinct profiles of patients existing beyond their diagnostic groups. This approach could be employed as an outcome measure in novel trial designs such as molecular pathology–driven and/or holistic management trials.

This study has several strengths. It compares HRQoL across several diseases recruited in the same cohort, thus reducing sampling and recruitment bias. The SF-36 HRQoL LPs were corroborated with EQ-5D scores in a subset of the cohort. This is a free and widely available tool that can be adopted in the clinical setting to measure HRQoL. Although disease-specific HRQoL scores incorporate components of HRQoL that are distinct to a given disease, the SF-36 is widely used and validated in CTDs and is a reliable measure of HRQoL that can be used across diseases [5–9].

This study has a number of limitations. First, there are no validated scoring systems to measure cross-disease activity in CTDs and disease-specific disease activity measures were not collected. In general, the patients in this cohort have relatively stable disease activity, therefore the external validity of these results in a more active cohort is unclear, as disease activity is likely to negatively impact HRQoL. Second, the data analysed in this study is cross-sectional, however, follow-up is presently under way. Prospective longitudinal data will address whether patient’s HRQoL may be improved by intervention in modifiable risk factors associated with a poor HRQoL LP, such as smoking cessation or weight reduction. Furthermore, the SSc-IIM spectrum group was composed of patients with SSc, IIM and overlap syndromes, due to the small numbers of patients with these diseases, which limits precision. In this analysis, patients were initially categorized by their physician diagnosis, which we chose as our ‘gold standard’. This was because all classification criteria sets come with the qualifier that they are not diagnostic criteria. It would be interesting in future analyses to consider what the optimal classification criteria for UCTD would be, and test this against the physician diagnosis in future studies of molecular taxonomy and clinical outcomes. Finally, we acknowledge that associates of HRQoL are multifactorial and many important covariates were not measured in this study, including damage scores and social factors (e.g. employment status, marital status and level of social support).

In conclusion, poor HRQoL is common in patients with CTDs and CTD patients can be clustered into distinct HRQoL subgroups that are not primarily driven by their specific diagnosis or autoantibody profile. We identified a number of key demographic, lifestyle and clinical factors associated with poor HRQoL in this population. These factors need to be addressed across the whole CTD spectrum as part of a holistic management approach aimed at improving overall patient outcomes.

## Supplementary Material

keac680_Supplementary_Data

## Data Availability

The LEAP Study is an ongoing prospective study and no data are available.
