# Supplementary material for: Determinants of health-related quality of life across the spectrum of connective tissue diseases using latent profile analysis: results from the LEAP cohort
Source: Rheumatology (Oxford). 2022 Dec 19;62(8):2673–82. doi: 10.1093/rheumatology/keac680 (PMC10393437; doi:10.1093/rheumatology/keac680)
Supplement: keac680_Supplementary_Data [file keac680_supplementary_data.docx]

**Supplementary Data S1; Latent Profile (LP) Analysis methods**

The eight continuous domains of the SF-36 questionnaire were included in the cluster analysis: physical function (PF), role physical (RP), bodily pain (BP), general health (GH), vitality (VT), social functioning (SF), role emotional (RE) and mental health (MH). These range from 0 to 100, with higher scores reflecting better HR-QoL.

LP analysis is a statistical method for identifying homogenous subgroups of individuals based on a set of continuous measured variables called indicators. Classification of individuals into latent classes is probabilistic and LP analysis allows for selection of the optimum numbers of classes (the optimum model), through comparison of model fit indices.

We fit the model with the latent class analysis (LCA) method using the Gaussian finite mixture model (GMM) clustering algorithm in R software (version 4.0.4) (using the “mclust” package, version 5.4.9) to identify clusters of patients who experienced distinct HR-QOL patterns.

GMM is probabilistic clustering technique that clusters data points based on the likelihood that they belong to a particular distribution. This means that data points within clusters were more similar to other data points in the same cluster and dissimilar to the data points in other clusters. In all models, variances were equated and covariances fixed to zero and missing values were imputed using methodology suggested by the SF-36 manual.

The Bayesian Information Criteria (BIC, figure 1) and Integrated Completed Likelihood Criterion (ICL, figure 2) were used to compare fits of models with different covariance structures and number of LPs. The values that are more negative are considered to fit poorer than values closer to 0. The Bootstrap Likelihood Ratio Test (BLRT) was performed which compares model fit between k-1 and k cluster models to see if an increase in profiles increases fit. This was performed using model VEE and 999 replications. Results for the above tests are summarised in table 1.

Each model was reviewed to ensure at least 5% of the population in each profile. We reviewed the classification plots for VEE3, VEE4 and VEE5 which showed optimal separation in the VEE3 (figure 3) profiling, comparative to VEE4 or VEE5.

The standardised mean domain score for VEE3 (figure 4), VEE4 (figure 5) and VEE5 (figure 6) were then plotted for visual inspection. We used hypothesis testing to compare variables, looking for significant differences between the clusters. In the four (VEE4) and five (VEE5) profile model, the separation of the profiles did not provide any more clinically meaningful findings; in VEE4 X1 and X3 show similar profiles and in VEE5 X1, X2 and X4 were similar, and felt to represent sub-sectioning one group. The three profile method selected (VEE3) provided distinct and clinically meaningful clusters with a minimum of 29 (9.4%) patients in each cluster.

Finally, this analysis was repeated on non-standardised data, this similarly found that VEE3 model was the optimal model, and results were similar with only 2 patients moving LP (one patient moves from LP2 to LP3 and one patient from LP1 to LP2).

| Model=VEE | Smallest profile % | BIC | ICL | BLRT test | BLRT p-value |
| --- | --- | --- | --- | --- | --- |
| 2 | 10.4 | -5363.09 | -5376.77 | 99.27766 | <0.001 |
| 3 | 9.4 | -5308.19 | -5347.79 | 112.23200 | <0.001 |
| 4 | 9.4 | -5297.92 | -5367.56 | 67.59475 | <0.001 |
| 5 | 9.7 | -5303.06 | -5390.33 | 52.19835 | <0.007 |

**Table S1:** Model fit test statistics. BIC, Bayesian information criteria; BLRT, Bootstrap likelihood ratio test; ICL, Integrated completed likelihood


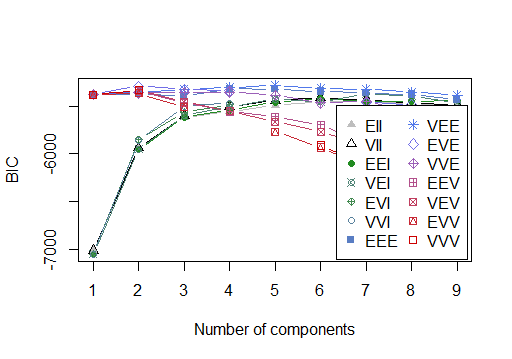


**Figure S1** - Bayesian information criteria (BIC) curve for each Gaussian model considered in the model-based latent profile analysis. The BIC plot shows each BIC value for each profile in which line graphs illustrate the different types of multivariate normal distributions integrated into the model per cluster. Each Gaussian model is illustrated with a different icon and a three-letter sequence. The letter sequence is a code for the geometric characteristics of volume, shape, and orientation. In the letter code, E means equal, V means varying across all clusters, and I specifies shape or orientation and is a special case of E.


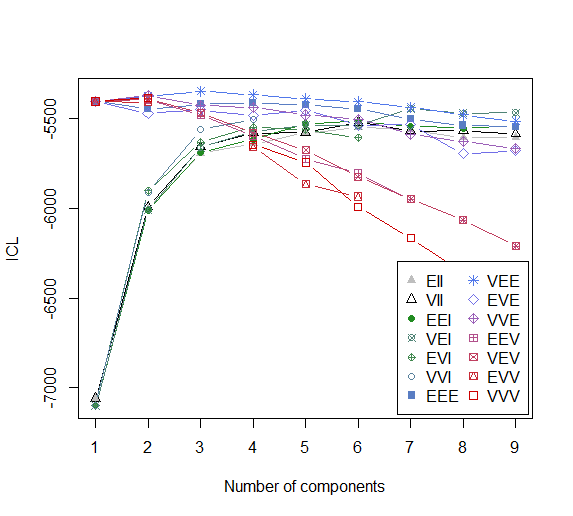


**Figure S2** - Integrated Completed Likelihood criterion (ICL) curve which adds a penalty on solutions with greater entropy or classification uncertainty. This plot shows each ICL value for each profile in which line graphs illustrate the different types of multivariate normal distributions integrated into the model per cluster. Each Gaussian model is illustrated with a different icon and a three-letter sequence as per the BIC graph.


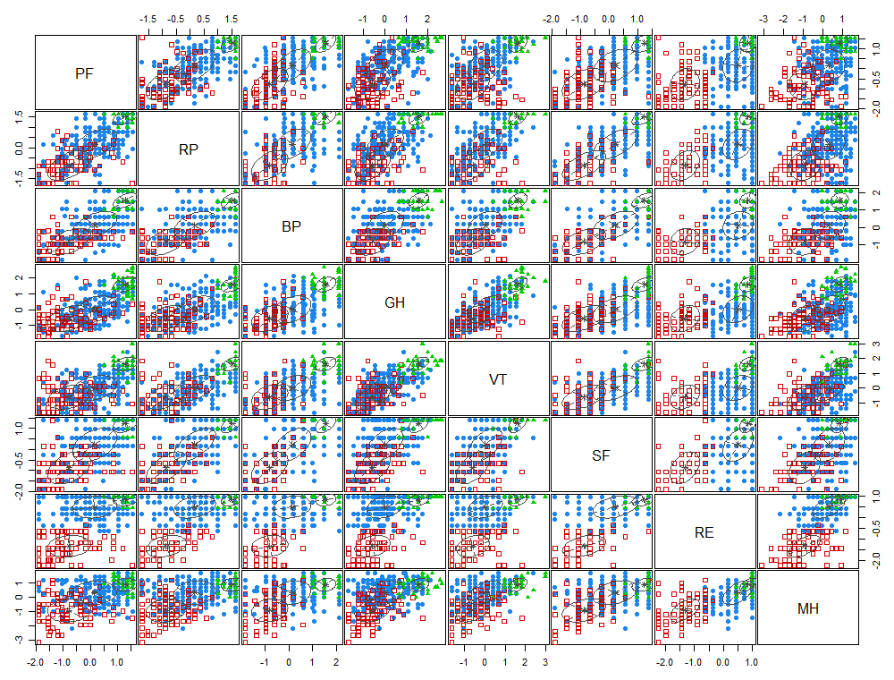


**Figure S3** - classification plot VEE3 show three LP in red, blue and green across the eight domains of the SF-36.


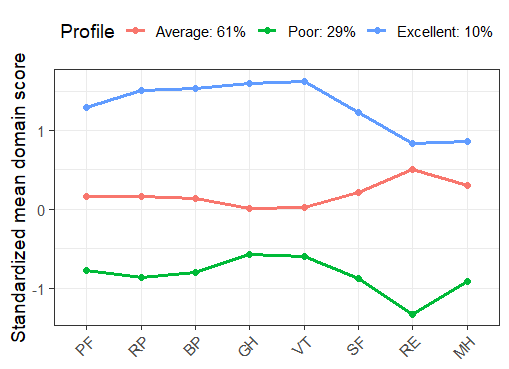


**Figure S4** - VEE3 - plotted standardised mean domain score for each of the three LP. BP, body pain; GH, general health; LP, latent profile; MH, mental health; PF, physical function; RE, role emotional; RP, role physical; SF, social function; VT, vitality.


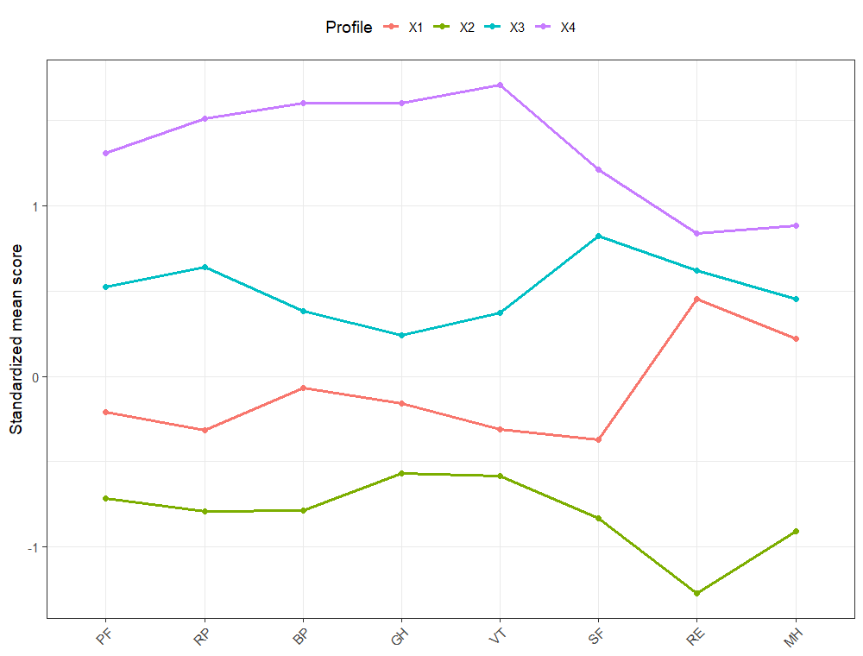


**Figure S5** - VEE4 plotted standardised mean domain score for each LP. Here X1 and X3 were felt to represent a similar LP of patients. BP, body pain; GH, general health; LP, latent profile; MH, mental health; PF, physical function; RE, role emotional; RP, role physical; SF, social function; VT, vitality.


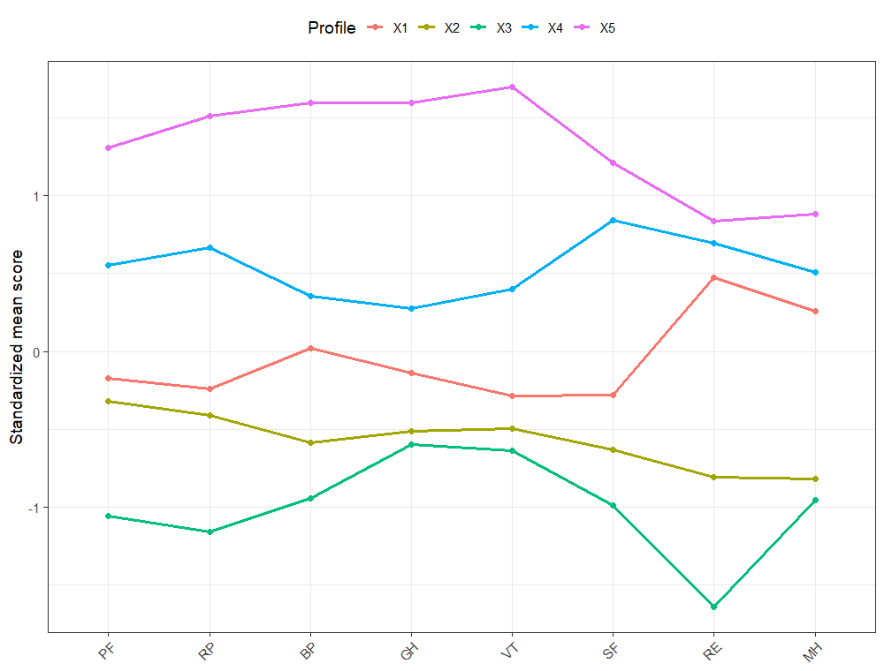


**Figure S6 -** VEE5 plotted standardised mean domain score for each LP. Here X1, X2, and X4 were felt to represent a similar LP of patients. BP, body pain; GH, general health; LP, latent profile; MH, mental health; PF, physical function; RE, role emotional; RP, role physical; SF, social function; VT, vitality

**Supplementary Data S2**

|  | **SSc** | **IIM** | **MCTD/ Overlap syndromes** |
| --- | --- | --- | --- |
|  | **(N=25)** | **(N=13)** | **(N=28)** |
| Female | 24 (96.0) | 13 (100) | 23 (82.1) |
| Age/ years | 61.8 (7.75) | 55.7 (7.15) | 49.7 (11.7) |
| Disease duration/ years | 10.0 (11.3) | 6.05 (6.40) | 10.5 (9.83) |
| Ethnicity |  |  |  |
| Caucasian | 23 (92.0) | 9 (69.2) | 22 (78.6) |
| Asian | 1 (4.0) | 0 (0) | 1 (3.6) |
| Black | 1 (4.0) | 2 (15.4) | 5 (17.9) |
| Other | 0 (0) | 2 (15.4) | 0 (0) |
| BMI | 30.6 (5.48) | 28.8 (4.65) | 25.6 (4.75) |
| Current smoker | 1 (4.0) | 2 (15.4) | 1 (3.6) |
| Ever smoker | 12 (48.0) | 6 (46.2) | 5 (17.9) |
| Fibromyalgia | 5 (20.0) | 0 (0) | 1 (3.6) |
| Anxiety or depression | 1 (4.0) | 0 (0) | 1 (3.6) |
| Osteoarthritis | 4 (16.0) | 1 (7.7) | 4 (14.4) |
| Hypermobility | 0 (0) | 1 (7.7) | 0 (0) |
| Sicca phenomenon | 10 (40.0) | 2 (15.4) | 9 (32.1) |
| Anti-Ro antibodies | 2 (8.0) | 4 (30.8) | 7 (25.0) |
| Number of co-morbidities |  |  |  |
| 0 | 6 (24.0) | 6 (46.2) | 8 (28.6) |
| 1-2 | 18 (72.0) | 6 (46.2) | 18 (64.3) |
| ≥3 | 1 (4.0) | 1 (7.7) | 2 (7.1) |

Table S2 Clinical characteristic by clinical diagnosis within IIM-SSc spectrum disorders diagnostic group by individual disease. Reported as N(%) or mean (SD). BP, body pain; general health; HR-QoL, health-related quality of life; IIM, idiopathic inflammatory myopathy; MCS, mental components score; MCTD, mixed connective tissue disease; MH, mental health; PCS, physical components score; PF, physical function; RE, role emotional; RP, role physical; SD, standard deviation; SF, social function; SSc, systemic sclerosis; VT, vitality.

|  | SLE | pSS | UCTD | SSC/ IIM/ overlap | All |
| --- | --- | --- | --- | --- | --- |
|  | (N=115) | (N=56) | (N=72) | (N=66) | (n=309) |
| Congestive cardiac disease | 1 (0.9) | 0 (0.0) | 2 (2.8) | 1 (1.5) | 4 (1.3) |
| Ischaemic heart disease | 3 (2.6) | 3 (5.4) | 4 (5.6) | 2 (3.0) | 12 (3.9) |
| Hypertension | 13 (11.3) | 5 (8.9) | 10 (13.9) | 5 (7.6) | 33 (13.7) |
| Thyroid disease | 16 (13.9) | 10 (17.9) | 10 (13.9) | 10 (15.2) | 46 (16.9) |
| Respiratory disease | 13 (11.3) | 7 (12.5) | 6 (8.3) | 16 (24.2) | 42 (13.6) |
| Peptic ulcer disease | 13 (11.3) | 10 (17.9) | 11 (15.3) | 14 (21.2) | 48 (13.5) |
| Chronic liver disease | 3 (2.6) | 0 (0.0) | 0 (0.0) | 0 (0.0) | 3 (3.0) |
| Chronic kidney disease | 11 (9.6) | 3 (5.4) | 5 (6.9) | 5 (7.6) | 24 (11.8) |
| Cancer | 5 (4.3) | 3 (5.4) | 1 (1.4) | 2 (3.0) | 11 (5.6) |
| Diabetes mellitus | 6 (5.2) | 0 (0.0) | 6 (8.3) | 3 (4.5) | 15 (6.9) |
| Transient ischaemic attack | 8 (7.0) | 1 (1.8) | 2 (2.8) | 0 (0.0) | 11 (8.6) |
| Stroke | 4 (3.5) | 0 (0.0) | 6 (8.3) | 0 (0.0) | 10 (4.2) |
| Addison’s disease | 2 (1.7) | 0 (0.0) | 1 (1.4) | 0 (0.0) | 3 (2.0) |
| Vitiligo | 1 (0.9) | 0 (0.0) | 1 (1.4) | 4 (6.1) | 6 (1.9) |
| atrial fibrillation | 1 (0.9) | 0 (0.0) | 1 (1.4) | 0 (0.0) | 2 (1.6) |
| Fibromyalgia | 15 (13.0) | 11 (19.6) | 10 (13.9) | 6 (9.1) | 42 (13.6) |
| Hypermobility | 5 (4.3) | 6 (10.7) | 12 (16.7) | 2 (3.0) | 25 (8.1) |
| Osteoarthritis | 6 (5.2) | 4 (7.1) | 2 (2.8) | 9 (13.6) | 21 (6.8) |
| Anxiety | 6 (5.2) | 2 (3.6) | 3 (1.2) | 1 (1.5) | 12 (3.9) |
| Depression | 8 (7.0) | 4 (7.1) | 3 (4.2) | 1 (1.5) | 16 (5.2) |

Table S3 – Comorbidities across connective tissue disease groups, N(%). The overlap proportion of this disease subgroup included patients with MCTD (n=23, 82%), those with an SSc-IIM overlap syndrome (n=3, 11%) and those with a lupus-IIM-SSc overlap (n=2, 7%). IIM, idiopathic inflammatory myopathy; MCTD, mixed connective tissue disease; pSS, primary Sjögren’s syndrome; SLE, systemic lupus erythematosus; SSc, systemic sclerosis; UCTD, undifferentiated connective tissue disease

|  | SLE  (N=115) | pSS  (N=56) | UCTD  (N=72) | SSc-IIM overlap  (N=66) | SSc  (N=25) | IIM  (N=13) | Overlap  (N=28) |
| --- | --- | --- | --- | --- | --- | --- | --- |
| SF-36 domain scores |  |  |  |  |  |  |  |
| **PF** | 58.2 (28.7) | 51.8 (29.4) | 52.7 (29.7) | 54.8 (28.6) | 47.6 (28.8) | 51.2 (31.6) | 63.0 (25.9) |
| **RP** | 54.1 (28.6) | 46.0 (30.9) | 49.2 (30.9) | 53.8 (29.5) | 47.8 (28.1) | 48.1 (33.0) | 61.8 (28.0) |
| **BP** | 48.6 (24.7) | 42.9 (23.3) | 41.5 (26.6) | 52.1 (26.7) | 50.9 (28.7) | 46.4 (29.4) | 55.9 (23.8) |
| **GH** | 36.6 (22.1) | 38.6 (24.2) | 33.1 (20.6) | 42.5 (21.7) | 41.7 (22.3) | 41.9 (22.5) | 43.5 (21.6) |
| **VT** | 37.1 (20.4) | 31.1 (20.5) | 30.1 (22.3) | 41.9 (22.1) | 36.9 (24.5) | 44.7 (19.4) | 45.1 (21.1) |
| **SF** | 59.2 (30.1) | 51.8 (28.6) | 53.8 (31.4) | 64.4 (31.2) | 60.0 (36.1) | 61.5 (24.7) | 69.6 (29.4) |
| **RE** | 70.9 (30.7) | 64.4 (34.0) | 70.7 (30.1) | 71.2 (30.7) | 62.3 (33.6) | 71.8 (35.3) | 78.9 (23.9) |
| **MH** | 66.0 (19.9) | 61.7 (22.3) | 64.4 (20.8) | 68.8 (21.6) | 66.4 (25.4) | 63.5 (22.7) | 73.4 (16.9) |
|  |  |  |  |  |  |  |  |
| SF-36 composite scores |  |  |  |  |  |  |  |
| **MCS** | 46.5 (10.5) | 44.2 (12.6) | 45.7 (11.0) | 48.9 (11.4) | 46.9 (13.8) | 48.7 (9.92) | 50.8 (9.73) |
| **PCS** | 31.9 (12.9) | 29.5 (12.4) | 28.8 (13.8) | 31.6 (12.8) | 29.6 (12.4) | 29.5 (15.0) | 34.3 (12.0) |
|  |  |  |  |  |  |  |  |
| EQ5D scores |  |  |  |  |  |  |  |
| **Index score** | 0.68 (0.31) | 0.54 (0.33) | 0.59 (0.30) | 0.69 (0.28) | 0.62 (0.29) | 0.75 (0.24) | 0.71 (0.35) |
| **VAS** | 53.8 (32.2) | 55.6 (24.8) | 60.4 (26.2) | 63.6 (24.7) | 65.5 (19.6) | 61.0 (28.1) | 64.7 (30.9) |

Table S4 - SF-36 and EQ5D scores across CTDs, to the right are the scores for IIM, SSc and overlap/MCTD groups. Data presented as mean (SD). BP, body pain; EQ5D, EuroQol-5D; GH, general health; IIM, idiopathic inflammatory myopathy; MCS, mental components score; MCTD, mixed connective tissue disease; MH, mental health; PCS, physical components score; PF, physical function; pSS, primary Sjögren’s syndrome; RE, role emotional; RP, role physical; SD, standard deviation; SF, social function; SLE, systemic lupus erythematosus; SSc, systemic sclerosis; UCTD, undifferentiated connective tissue disease; VAS, visual analogue score; VT, vitality.


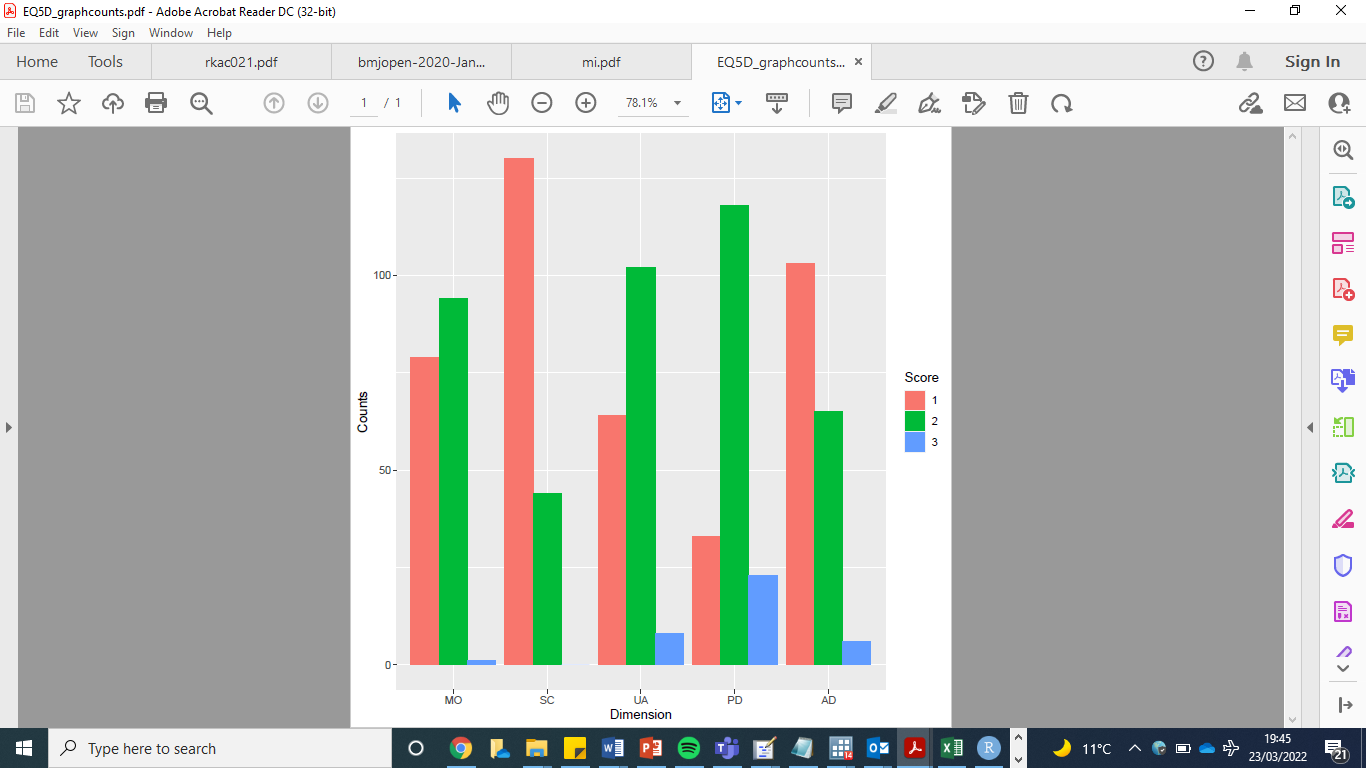


Figure S7 - EQ5D scores in SARDs cohort (n=174) 1=‘no problems’, 2=‘moderate problems’, 3=‘severe problems’. AD, anxiety and depression; MO, mobility; PD, pain and discomfort; SC, self-care; UA, usual activity
